# Supplementary material for: Content-rich biological network constructed by mining PubMed abstracts
Source: BMC Bioinformatics. 2004 Oct 8;5:147. doi: 10.1186/1471-2105-5-147 (PMC528731; doi:10.1186/1471-2105-5-147)
Supplement: Additional File 2 — The original results of the above study (non-essential files are deleted to keep the file size under the limit set by BMC bioinformatics). [file 1471-2105-5-147-S2.bz2 › chilibotAdditionalFile2/dip30/540ID9211894E10893/html/AR_FOS.html]

 


 **AR** and **FOS** 
  
Found 62 abstracts in PubMed, retrieved 30.  
 

 What does Google say? 
 PDF only 
| .edu only 

---

**Interactive relationship** (e.g. stimulation, inhibition, etc)

**Inhibitory relationship**- Compared with MR rats, pup exposed  **AR**  rats engaged in less pup licking, and all  **AR**  rats showed significant reductions in c  **fos**  immunoreactivity in the medial preoptic area and the parietal and piriform cortices.  Ref: 12492299 Behav Neurosci, 2002
- Pre treatment with the selective beta 3  **AR**  antagonist SR59230A resulted in a significant decrease in the number of  **Fos**  positive cells in all those areas compared with rats treated with CL316243 alone.  Ref: 10963888 Neurosci Lett, 2000
- In addition to its ability to activate transcription from androgen response elements,  **AR**  can inhibit activator protein 1 AP 1 activity, composed of Jun and  **Fos**  oncoproteins, in a ligand dependent manner.  Ref: 9822653 J Biol Chem, 1998
**Neutral relationship**- Neurons in these brain areas are rich in androgen receptors  **AR**  and express  **FOS**  immunoreactivity in response to mating.  Ref: 12689608 Psychoneuroendocrinology, 2003
- The  **AR**  min group showed the greatest difference in  **Fos**  lir compared with the MR groups.  Ref: 12492299 Behav Neurosci, 2002
- **Fos**  was also localized in subsets of  **AR**  ir neurons within the L5, L6, and S1 segments of the spinal cord.  Ref: 9647938 Horm Behav, 1998
- **Fos**  was highly localized in subsets of  **AR**  ir neurons within the medial preoptic nucleus, bed nucleus of the stria terminalis, dorsomedial nucleus of the amygdala, and central tegmental field.  Ref: 9647938 Horm Behav, 1998
- Spinal cord concentrations of  **AR**  ir and  **Fos**  ir neurons were greatest in Lamina X, and the vast majority of  **Fos**  ir neurons in the dorsal part of Lamina X were also  **AR**  ir.  Ref: 9647938 Horm Behav, 1998
- c  **Fos**  dimerization with c Jun represses c Jun enhancement of androgen receptor  [ **AR** ]  transactivation.  Ref: 9867253 Endocrine, 1998

**Non-interactive relationship** (e.g. studied together, co-existance, homology, etc.)

- After testing, brains and spinal cords were examined for  **AR**  and  **Fos**  immunoreactivity ir.  Ref: 9647938 Horm Behav, 1998
- There is now evidence for a critical cell signaling pathway, regulated by c  **fos**  expression, necessary for castration induced apoptosis, as well as evidence that this signaling initiates an abrupt and transient alteration in the synthesis of fas antigen, p53, bax and bcl 2 proteins in the androgen receptor  [ **AR** ]  expressing prostate epithelial cells, the cellular compartment that appears to be the most affected by castration.  Ref: 10322394 Trends Endocrinol Metab< MedlineTA>Trends Endocrinol Metab, 1998
- We report that, identically to 17beta estradiol or dihydrotestosterone, but differently from raloxifene, estren alters the activity of Elk 1, CCAAT enhancer binding protein beta C EBPbeta, and cyclic adenosine monophosphate response element binding protein CREB, or c Jun c  **Fos**  by an extranuclear action of the ER or  **AR** , resulting in activation of the Src Shc ERK pathway or downregulation of JNK, respectively.  Ref: 12782668 J Clin Invest, 2003
- PG21 anti  **AR**  and anti c  **fos**  primary antibodies were visualized by fluorescence microscopy using cyanine conjugated and fluorescein conjugated secondary antibodies.  Ref: 9647938 Horm Behav, 1998
- This study was designed to determine the extent to which  **Fos**  immunoreactivity induced either by mating or noncontact sociosexual interaction and androgen receptor  [ **AR** ]   **AR**  immunoreactivity are colocalized in brain and spinal cord of male rats.  Ref: 9647938 Horm Behav, 1998
- The alpha 2  **AR**  agonists, clonidine and UK14304 also had no effect on basal IEG levels, while blockade of alpha 2 ARs by methoxyidazoxan significantly increased NGFI A and c  **fos**  expression, but decreased c jun mRNA levels.  Ref: 11072104 Brain Res Mol Brain Res, 2000
- Here, we report that alpha 1 B ARs inhibit the activities of alpha 1A ARs in neonatal rat myocardium so that the inactivation of alpha 1 B ARs by chloroethylclonidine CEC potentiated the effects of nonselective alpha 1  **AR**  agonist phenylephrine PE on myocardial protein synthesis and early gene c  **fos**  and c jun expression.  Ref: 9655894 J Pharmacol Exp Ther, 1998
- Effect of prenatal androgen receptor  [ **AR** ]  antagonist or aromatase inhibitor on sexual behavior, partner preference and neuronal  **Fos**  responses to estrous female odors in the rat accessory olfactory system.Many socially relevant odors are detected in rodent species by the vomeronasal organ and subsequently processed by the accessory olfactory system.  Ref: 11897260 Physiol Behav, 2002
- We have conducted experiments investigating the effects of i.c.v. administration of the selective beta 3  **AR**  agonist CL316243 on  **Fos**  expression to determine whether beta 3  **AR**  stimulation affects neurones within specific brain nuclei.  Ref: 10963888 Neurosci Lett, 2000
- We have previously reported an indirect mechanism by which c Jun is able to regulate transcription by stimulating androgen receptor  [ **AR** ]  transactivation in the absence of c  **Fos**  or any apparent DNA binding.  Ref: 9591784 Oncogene, 1998
- Although the AP 1 c Jun c  **Fos**  system and NcoR have been reported to interact with and repress the activity of some steroid receptors, c Jun, c  **Fos** , c Jun c  **Fos** , nor NcoR function was consistently affected by the absence or presence of residues 628 646, implying that the  **AR**  hinge region exerts its silencing effects in a manner independent of these corepressors.  Ref: 11102454 J Biol Chem, 2001
- Moreover, the brain dopamine and serotonin balance, but not norepinephrine turnover, was dependent on alpha2C  **AR**  expression, and the expression of c  **fos**  and junB mRNA was increased in alpha2C KO mice.  Ref: 10523817 Mol Psychiatry, 1999
- Since activator protein 1 AP 1, a heterodimer of c jun and c  **fos**  proteins, is known to inhibit binding of  **AR**  to ARE by protein protein interaction with  **AR** , the findings in the present study suggest a possible involvement of AP 1 in the antiandrogenic effects of PAHs acting as AhR agonists.  Ref: 12799773 Arch Toxicol, 2003
- **AR**  ir and  **Fos**  ir were visualized with fluorescence microscopy using cyanine conjugated and fluorescein conjugated secondary antibodies.  Ref: 10333272 J Comp Neurol, 1999
- After experiencing social stress the brain area within the medial preoptic area was removed for analysis of c  **fos**  and androgen receptor  [ **AR** ]  mRNA expression.  Ref: 12131378 J Urol, 2002
- Brains and spinal cords were examined for FG transport,  **AR**  immunoreactivity  **AR**  ir, and  **Fos**  immunoreactivity  **Fos**  ir.  Ref: 10333272 J Comp Neurol, 1999
- This study examined if mating induced  **Fos**  expression a measure of neuronal activation and androgen receptors  **AR**  are colocalized in brain and spinal cord neurons which project to the CTF.  Ref: 10333272 J Comp Neurol, 1999
- ... inhibition of c  **Fos** , c Jun, HB EGF,  **AR** , and VEGF mRNA by this compound was incomplete 41 65%.  Ref: 12016209 J Biol Chem, 2002
